# Transverse Strains in Muscle Fascicles during Voluntary Contraction: A 2D Frequency Decomposition of B-Mode Ultrasound Images

**DOI:** 10.1155/2014/352910

**Published:** 2014-09-28

**Authors:** James M. Wakeling, Avleen Randhawa

**Affiliations:** Department of Biomedical Physiology and Kinesiology, Simon Fraser University, Burnaby, BC, Canada V5A 1S6

## Abstract

When skeletal muscle fibres shorten, they must increase in their transverse dimensions in order to maintain a constant volume. In pennate muscle, this transverse expansion results in the fibres rotating to greater pennation angle, with a consequent reduction in their contractile velocity in a process known as gearing. Understanding the nature and extent of this transverse expansion is necessary to understand the mechanisms driving the changes in internal geometry of whole muscles during contraction. Current methodologies allow the fascicle lengths, orientations, and curvatures to be quantified, but not the transverse expansion. The purpose of this study was to develop and validate techniques for quantifying transverse strain in skeletal muscle fascicles during contraction from B-mode ultrasound images. Images were acquired from the medial and lateral gastrocnemii during cyclic contractions, enhanced using multiscale vessel enhancement filtering and the spatial frequencies resolved using 2D discrete Fourier transforms. The frequency information was resolved into the fascicle orientations that were validated against manually digitized values. The transverse fascicle strains were calculated from their wavelengths within the images. These methods showed that the transverse strain increases while the longitudinal fascicle length decreases; however, the extent of these strains was smaller than expected.

## 1. Introduction

Muscle fibres are nearly incompressible [1] and so must increase in girth as they shorten. This transverse expansion requires that the fibres in pennate muscle rotate to greater pennation angles during shortening to ensure they still pack together in close proximity [2, 3]. Due to these fibre rotations, the fibres shorten at a lower velocity than the muscle belly in a process known as muscle belly gearing [4–6], promoting a greater force and power production from the muscle. Understanding how muscle fibres change shape during contraction will elucidate how structural mechanisms affect the functional output of muscle.

It is commonly assumed that whole muscles maintain a constant volume during contraction, in a similar manner to their constituent fibres. Many muscle models are one-dimensional and thus assume constant thickness of the muscle belly [3, 7–10]. Some studies have modelled muscle as 2D structures but have implemented a constant area assumption [6, 11–13], and other studies of 3D properties implement constant or nearly incompressible volumes [14–17]. However, whole muscle may change volume to a greater extent than fibres, due to a variable amount of blood that may pool and be pumped out from the muscle, through the action of the muscle contraction [18]. Additionally, recent imaging studies have shown that muscle varies in thickness in a complex and muscle-specific manner. Muscle thickness is not constant during contraction and can vary for both isometric [3] and dynamic [5, 6, 19] contractions.

The belly gearing within a muscle can vary according to the mechanical demands of the contraction [5, 6, 19], and so the fibres may exist at a range of different pennation angles for a given fibre length: it has been suggested that this variability is related to the load and stretch of connective tissue such as aponeurosis [6, 15, 19]. Currently we do not know how the changes in transverse fibre dimensions relate to dynamic changes in fibre geometry, belly gearing, and thus the functional output of the muscle.

Recent developments in muscle imaging have allowed the longitudinal properties of muscle fibres to be determined. Diffusion-tensor MRI can identify the longitudinal direction of muscle fibres [20–24] allowing fibres to be tracked and their length calculated. However, MRI imaging necessitates prolonged, isometric contractions and thus is not suitable for dynamic studies of gearing. B-mode ultrasound imaging of muscle allows the fascicles to be imaged at faster rates, and a range of automated approaches have been used to quantify the fascicle lengths and even curvatures during dynamic contractions [25–28]. Neither these MRI nor ultrasound techniques have allowed the width of the fascicles, or a measure of their transverse expansion, to be quantified. However, muscle fascicles appear as nearly parallel, repeating bands within B-mode ultrasound images and the width of these bands can potentially be extracted by frequency decomposition of the images. The purpose of this study was to develop a method to extract information on the spatial frequencies of the fascicular structure within the muscle belly from B-mode ultrasound images and to relate this information to the fascicle size, shape, and orientations during muscle contraction.

## 2. Methods

### 2.1. Subjects

Six male subjects took part in this study (age 28.8 ± 5.5 years; mass 78.3 ± 6.2 kg; height 178 ± 2.3 cm; mean ± SD). All subjects provided informed consent in accordance with requirements from the University Office of Research Ethics.

### 2.2. Data Collection

Images were acquired from the medial gastrocnemius (MG) and lateral gastrocnemius (LG) of the right leg during ankle plantarflexion contractions. Subjects were seated on a dynamometer (System 3, Biodex, New York, USA) with their knee held at 135°, their shank horizontal, and their foot secured to a footplate on the dynamometer. The central axis of the dynamometer was aligned to meet the axis through the medial and lateral maleoli. Subjects performed cyclic ankle extensions against isotonic loads, in time to the beat of a metronome. The dorsiflexion torque was limited to 0.5 N m, and three plantarflexion conditions were presented (5@0.42, 25@0.35, and 5@16: torque [N m] @ cycle frequency [Hz]); each condition had a 15° range of motion from 5 to 20° plantarflexion. Each trial consisted of 10 cycles of contraction, from which the middle 5 were analyzed.

The MG and LG muscle bellies were imaged using 128-element (60 mm width) linear array B-mode ultrasound probes (Echoblaster 128, Telemed, Lithuania), scanning at 40 Hz. The probes were aligned to the fascicle planes to obtain nearly continuous lines for the fascicles in each image. The probes were secured to the leg using custom mounts with adhesive and elasticized bandages. The probes were measured from the MG and LG simultaneously,and were synchronized to the position *P* and torque data *T* from the dynamometer (recorded at 1000 Hz: USB-6229, National Instruments, Austin, TX, USA).

### 2.3. Image Analysis

**Figure 1:**
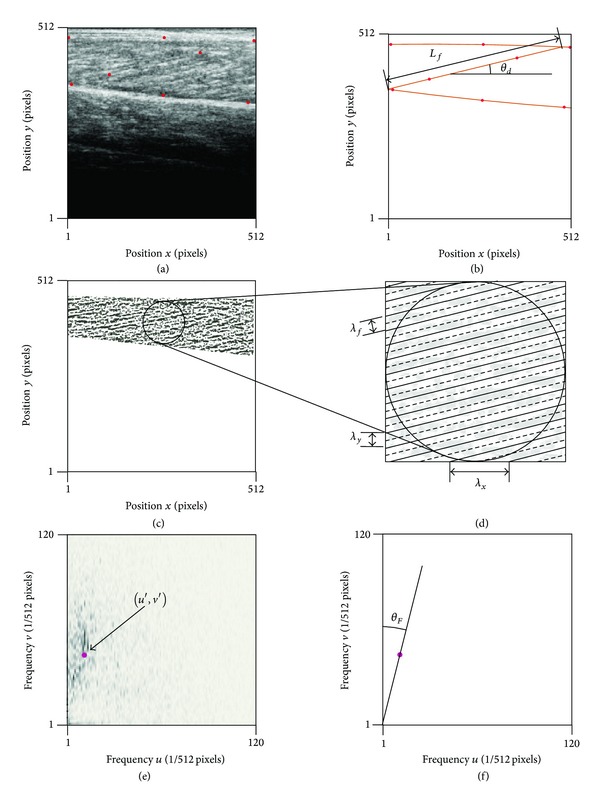
*The Analysis Method*. Coordinates on the superficial and deep aponeuroses and a representative fascicle were manually digitized (red points) for each ultrasound image (a) and used to calculate the fascicle length *L*_*f*_ and inclination angle *θ*_*d*_ relative to the *x*-axis (b). Fascicles were enhanced using multiscale vessel enhancement filters and the region of interest was determined between the aponeuroses (c). The repeating fascicular nature is described by its transverse wavelength *λ*_*f*_, which can be resolved into wavelengths *λ*_*u*_ and *λ*_*v*_ that are in the *x*- and *y*-directions, respectively (d). The spatial frequencies *u* and *v* were determined using a discrete Fourier transform, and the major spatial frequencies characterized by the moment of frequency (*u*′, *v*′) (e). The inclination angle *θ*_*F*_ was were determined from the major spatial frequencies (f), and the wavelengths *λ*_*u*_ and *λ*_*v*_ were calculated from these frequencies.

Each ultrasound frame formed a square image *f*(*x*, *y*) of *N* = 512 pixels per side with each greyscale pixel indexed by its *x*- and *y*-coordinate (Figure 1). Images were manually digitized (ImageJ software, NIH, Maryland, USA) to identify three coordinates on the superficial aponeurosis, three coordinates on the deep aponeurosis, and two coordinates on a representative fascicle. Aponeuroses were described using second-order polynomials that were fit to both the superficial and the deep coordinates using least-squares minimization, and the muscle belly thickness *L*_*y*_ was calculated as the mean distance between the aponeuroses. The fascicle inclination *θ*_*d*_ within the fascicle plane was given by the angle between the *x*-axis and the vector between the two digitized fascicle points. The fascicle length *L*_*f*_ was given by the length of the linear line passing through the fascicle coordinates that intersected the best-fit linear lines through the superficial and deep aponeuroses.

Each image was filtered using multiscale vessel-enhancement filtering. This method enhances the tubular structures in the image that are formed by the fascicles [29] and is capable of resolving tubular structures of different radii and has previously been applied to B-mode ultrasound images [25, 26, 30]. Here we followed Rana and coworkers [25] by using scales of 1.5, 2, 2.5, and 3. The region of interest was taken as the area of muscle tissue within the filtered image that was bounded by the aponeuroses, and a strip, 10 pixels wide, was removed inside the aponeuroses to ensure that the region of interest contained no features that were aligned with the aponeuroses.

Muscle fascicles appear as dark lines in the image and connective tissue between the fascicles appears as bright structures that parallel the fascicles [25]. The striped nature of the fascicles is enhanced and retained within the filtered image and is characterized by the spatial frequency of the stripes. The spatial frequencies *F*(*u*, *v*) of the filtered image *f*(*x*, *y*) were determined by a 2D discrete Fourier analysis of the region of interest, where

(1) F(u,v) =1N∑x=0N−1∑y=0N−1f(x,y)  ×[Cos⁡(2π(ux+vy)N)+jSin⁡(2π(ux+vy)N)],j=−1.

The amplitude spectra for the region of interest describe the amplitudes of the pixel intensities across a range of frequencies:

(2) |F(u,v)|=Re2(u,v)+Im2(u,v).

This is reduced to a single frequency value for each direction (*u*′ and *v*′) using the *m*th moment of frequency:

(3) u′=∑u=b1b2∑v=b1b2[|F(u,v)|mu]∑u=b1b2∑v=b1b2|F(u,v)|m,v′=∑v=b1b2∑u=b1b2[|F(u,v)|mv]∑v=b1b2∑u=b1b2|F(u,v)|m.

The moments of frequency were calculated across the frequency range of *b*_1_ = 4 to *b*_2_ = 120, and this contained more than 99% of the power of the amplitude spectra.

The wavelengths for the fascicle stripes *λ*_*x*_ and *λ*_*y*_ were given by

(4) $$\begin{array}{c} \lambda_{x}=\frac{1}{u^{\prime }},\;\;\lambda_{y}=\frac{1}{v^{\prime }}. \end{array}$$

The dominant repeating structure within the region of interest is given by the muscle fascicles. For large inclinations (≈90°) *λ*_*x*_ would be small and *λ*_*y*_ would be large; conversely, for small inclinations (≈0°) *λ*_*x*_ would be large and *λ*_*y*_ would be small. The fascicle inclination *θ*_*F*_ (relative to the *x*-direction) can be determined from the Fourier analysis as follows:

(5) $$\begin{array}{c} \theta_{F}=\text{ArcTan}\left(\frac{\lambda_{y}}{\lambda_{x}}\right). \end{array}$$

The best value for *m* was determined by comparing *θ*_*d*_ and *θ*_*F*_ for 1 ≤ *m* ≤ 8 (see Statistics section below).

The wavelengths *λ*_*x*_ and *λ*_*y*_ reflect the dominant characteristics of the repeated fascicles in the region of interest. They provide information not only on the fascicle inclination, but also on the wavelength of the “fascicle stripes” *λ*_*f*_, that is, the wavelength of the stripes in a transverse direction across the fascicles:

(6) λf=λxSin⁡θF.

The muscle belly thickness *L*_*y*_, fascicle length *L*_*f*_, and the wavelength *λ*_*f*_ were normalized by their respective means that occurred throughout the five contraction cycles to yield normalized terms ${\hat{L}}_{y}$, ${\hat{L}}_{f}$, and ${\hat{\lambda }}_{f}$, respectively.

Pixel brightnesses in each ultrasound image are a measure of the echogenicity of the material being scanned. It is possible that, as the muscle expands in a transverse direction, there is an uneven expansion of fascicular and connective tissue. This would result in a change in the distribution of pixel brightness within the region of interest. This possibility was examined by quantifying the mean pixel brightness *B*_*p*_ within the region of interest.

### 2.4. Statistics

The best value for *m* was determined from the correlation coefficient *r* and the root-mean-square error (RMSE) between *θ*_*d*_ and *θ*_*F*_ for each contraction sequence. The effect of *m* on *r* and RMSE was determined with ANOVA with subject (random), muscle, and condition as factors (Minitab v16, Minitab Inc., State College, PA, USA).

For each condition the time *ω* was normalized to each contraction (0 to 360°), with 0° occurring at the midpoint of each dorsiflexion movement. The parameters *P*, *T*, ${\hat{L}}_{f}$, ${\hat{L}}_{y}$, *θ*_*d*_, *θ*_*F*_, ${\hat{\lambda }}_{f}$, and *B*_*p*_ were each described by a Fourier series of the form

(7) c1+a1Sin⁡(ϕ1+ω)+a2Sin⁡(ϕ2+2ω),

where the coefficients *c*_1_, *a*_1_, and *ϕ*_1_ describe the mean value, the amplitude, and the phase for the first harmonic. The effect of subject (random), muscle, condition, parameter, and muscle-by-parameter interaction on these Fourier coefficients was determined using ANOVA.

Statistical tests were considered significant at the *α* = 0.05 level. Mean values are reported as mean ± standard error of the mean.

## 3. Results

**Figure 2:**
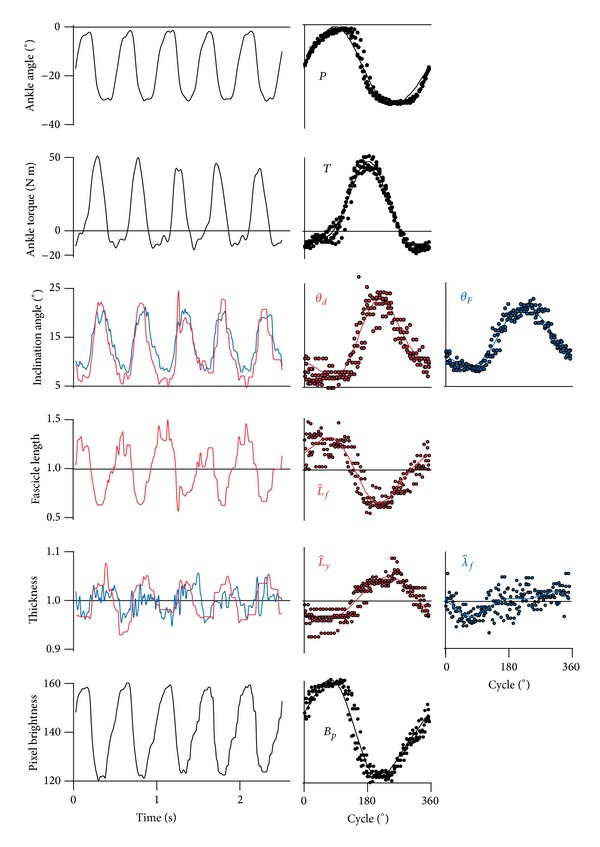
*Muscle Structural Data for the Lateral Gastrocnemius during One Set of Ankle Plantarflexions*. The first column shows the parameters changing over five contraction cycles. The middle and right columns show these data expressed as a percentage of the contraction cycle and show individual points as well as the smooth model calculated from Fourier series. Parameters determined from manual digitization are shown in red, and parameters determined following the discrete Fourier transform are shown in blue. ${\hat{\lambda }}_{f}$ was calculated with *m* = 5.

Subjects performed a series of isotonic plantarflexions (Figure 2), with the ankle plantarflexor torque increasing during each plantarflexion. The fascicle length within the medial and lateral gastrocnemius shortened during each plantarflexion, and this coincided with an increase in the inclination angle of the fascicles. During fascicle shortening the thickness of both the fascicles and muscle belly increased, with the relative increases in the muscle belly thickness being greater than those for the fascicles. During each contraction cycle, the pixel intensity within the region of interest varied, with the lowest intensities occurring when the fascicles were shortest but thickest.

**Figure 3:**
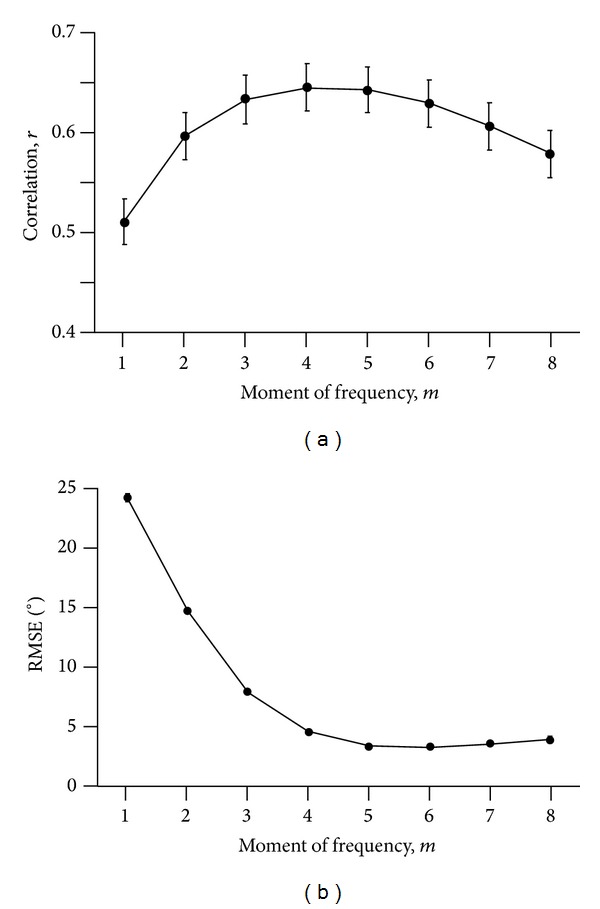
*Error Analysis for the Selection of the Moment of Frequency*, *m*. The correlation between the inclination angle determined by manual digitization, *θ*_*d*_, and the inclination angle determined from the discrete Fourier transform, *θ*_*F*_, is shown for different values of *m* (a). The root-mean-square error between these terms is shown in (b). Points show the mean ± SEM (*n* = 99).

The estimates of the inclination angle based on the Fourier transform were dependent on the moment of frequency, *m*, selected. There was no significant effect of *m* on the correlation between the inclination angle determined by manual digitization, *θ*_*d*_, and the inclination angle determined from the discrete Fourier transform, *θ*_*F*_; however, there was a significant effect of *m* on the root-mean-square error between these values (Figure 3). A value of *m* = 5 resulted to be close to the greatest correlation and the lowest RMSE and so was selected for further analysis. When considered across all subjects, muscles, and contraction conditions, the RMSE for *m* = 5 was 3.4°. The error between the two measures of inclination was partly due to the smaller amplitude of change in inclination for the *θ*_*F*_ than for *θ*_*d*_.

**Figure 4:**
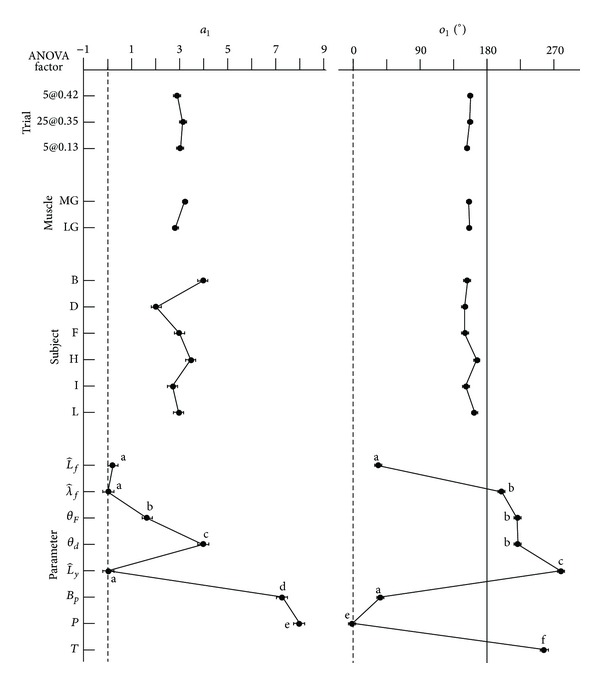
*Main Effects Determined by ANOVA*. The effects of the experimental factors on the coefficients *a*_1_ and *o*_1_ for the first harmonic in the Fourier series are shown. Points show the least-square mean ± SEM as determined by the ANOVA. *a*_1_ for torque was not tested due to its magnitude being much larger than the other variables. Letters next to the symbols indicate parameters that were not significantly different from each other, as calculated in a post hoc Tukey analysis.

The ANOVA showed there was a significant effect of the muscle, subject, parameter, and muscle-by-parameter interaction on the amplitude of the cyclic changes, *a*_1_. The main effects from the ANOVA (Figure 4) showed that the *a*_1_ for MG was 0.41 greater than for LG. The interaction effect showed that *a*_1_ for *θ*_*d*_ for the MG was greater than for the LG; however, this effect was not seen for *θ*_*F*_. The magnitude of the parameter effects on *a*_1_ can be seen in Tables 1 and 2.

The ANOVA showed there was a significant effect of the subject, parameter, and muscle-by-parameter interaction on the phase of the cyclic changes, *o*_1_. The interaction effect showed that *o*_1_ for ${\hat{\lambda }}_{f}$ was slightly smaller and for ${\hat{L}}_{y}$ was slightly larger for the MG than for the LG: in other words, there was a greater phase difference between the cycles of fascicle thickness and muscle belly thickness for the MG than for the LG. The magnitude of the parameter effects on *a*_1_ can be seen in Tables 1 and 2: there was no significant difference in the phase difference between ${\hat{L}}_{y}$, *θ*_*d*_, and *θ*_*F*_. When the phase for fascicle length was offset by 180° (*o*_1_ + 180°) there was no significant difference between its phase and those for ${\hat{L}}_{y}$, *θ*_*d*_, and *θ*_*F*_, and so the timing of fascicle length shortening exactly matches the increases in fascicle thickness.

## 4. Discussion

This study shows that there is information within B-mode images of the muscle bellies that has spatial frequencies that change in a cyclical manner during repeated contractions. These spatial frequencies are due to the fascicular (or vessel-like) structures within the muscle that were resolved by the multiscale vessel enhancement filtering [29]. 2D information from the images was retained by the 2D discrete Fourier transform of the images and allowed the inclination angle of the muscle fascicles to be determined, *θ*_*F*_: this is a feature of the fascicles that could be validated against the manually determined inclination angles, *θ*_*d*_ (Figure 3).

A perfect match between *θ*_*d*_ and *θ*_*F*_ should not be expected. When ultrasound images are manually digitized, the inclination angles tend to reflect the dominant fascicle features within the image [25]. However, there is variation of fascicle orientations across each image [26] and sometimes nonfascicular features that may also occur in the image, and these features would influence the spatial frequencies determined by automated methods that consider the whole region of interest [25] such as the discrete Fourier transform as used in this study. The accuracy in these automated approaches can be maximized by careful selection of images that contain minimal nonfascicular structures [30] or by masking the undesired features within the region of interest. The inclinations *θ*_*d*_ and *θ*_*F*_ in this study measured the angles between the fascicles and the *x*-axis of the ultrasound images. By contrast, pennation angles measured in previous ultrasound studies are defined in different ways, for example, the angle between the fascicle and the superficial aponeurosis, or the deep aponeurosis or the mean direction of the superficial and deep aponeuroses [3, 31–33]. In this study, the *θ*_*F*_ was approximately 13° and 8° for the MG and LG, respectively, for an ankle plantarflexion angle of 5° (calculated from data in Tables 1 and 2), and these are approximately 5° smaller than the pennations reported for seated subjects in the same dynamometer [6].

The transverse wavelength of the fascicle strains ${\hat{\lambda }}_{f}$ changed in a cyclical manner, in time with the fascicle shortening (Tables 1 and 2; Figure 4). As the fascicles shorten their transverse strain increased. The mean pixel brightness also decreased as the transverse strain increased (Tables 1 and 2; Figures 2 and 4), indicating a greater proportion of darker elements in the image for higher ${\hat{\lambda }}_{f}$. Within the muscle bellies, the fascicles and connective tissue have different echogenicities, with the fascicles appearing darker. It is possible that the increase in *B*_*p*_ with increased ${\hat{\lambda }}_{f}$ indicates that the transverse strain in the muscle fascicles is underestimated by ${\hat{\lambda }}_{f}$ (that includes elements from both fascicles and connective tissue).

The transverse strain was calculated by resolving *λ*_*x*_ and *λ*_*y*_ into the transverse direction. In theory, these values could be resolved into the longitudinal direction to provide a measure of longitudinal fascicle strain; however, this is practically not possible. In an ideal ultrasound image, all the fascicles would appear as continuous lines between the two aponeuroses: these would have a longitudinal spatial frequency of less than 1/512 pixels and therefore would be beyond the resolution of the technique. In reality, fascicles appear as partial lines between the aponeuroses, and the exact length of each line segment is very sensitive to the exact orientation of the fascicles relative to the scanning plane. During contraction the fascicles can change their orientation relative to the scanning plane [30], and thus fluctuations in line-length would reflect their 3D orientation as well as the fascicle length and therefore preclude measurements of fascicle length using these methods.

Assuming that the muscle fibres (and presumably the fascicles) maintain a constant volume during contraction [1], then they must increase in girth as they shorten. If an additional assumption is made that the increase in girth for the muscle fascicles is radially symmetrical then the muscle fascicles should have a Poisson ratio of 0.43 for a longitudinal strain of 0.2, where the Poisson ratio is the ratio of the transverse strain/longitudinal strain. The Poisson ratio *ν* can be calculated from this study as the ratio of (*a*_1_ for ${\hat{\lambda }}_{f}$)/(*a*_1_ for ${\hat{L}}_{f}$). The mean *ν* from this study was 0.09 ± 0.01 and was thus much smaller than expected. As discussed above, it is possible that ${\hat{\lambda }}_{f}$ is an underestimate, leading to low *ν*. An alternative estimate for the transverse strain for the fascicles can be calculated from the manually digitized parameters. If it is assumed that the entire muscle belly consists of fascicles that are parallel to each other, then the distance between the aponeuroses must equal the width of the fascicles acting in parallel, adjusted by their inclination; thus the normalized fascicle thickness will equal ${\hat{L}}_{y}/\mathrm{Cos}\theta_{d}$. This alternative estimate of fascicle thickness yields a mean *ν* of 0.20 ± 0.02 that is still less than expected. Data from this study thus indicate that the increase in the transverse width of the fascicles does not meet that expected for isovolumetric muscle fibres that show radial symmetry in their expansion in girth. It will be necessary to investigate the fascicular expansion in the direction perpendicular to the scanning plane to identify the reasons for this discrepancy.

Changes to muscle belly thickness occur with changes in both fascicle thickness and fascicle rotations to different pennation angles [19], with the fascicle thickness and pennation angle being related to each other via intramuscular pressure, transverse forces, and compliance in connective tissues [15] such as aponeuroses and intramuscular connective tissue [34]. It is possible that the differences in whole-muscle bulging between MG and LG that have been reported in previous studies [3, 5, 6, 19, 32] may reflect differences in the direction of the transverse or perpendicular (out of plane) bulging of the fascicles, or due to differences in connective tissue properties and the tendency for the fascicles to rotate. In this study we found similar transverse expansion and Poisson ratio of the fascicles occurring in both the MG and LG (Tables 1 and 2), thus indicating that differences in the bulging of the muscle belly are caused more by differences in connective tissue properties and the tendency for the fascicles to rotate than by differences in the fascicle bulging* per se*.

This study describes a method to determine the transverse strain in the muscle fascicles during contraction and is the first study to describe these strains during dynamic and voluntary contractions. However, it should be noted that this methodological study has been constrained to a small set of contractions performed by male subjects: it will be important to understand how transverse bulging of the fascicles changes with both age and gender. Nonetheless, the results show that increases in transverse width are exactly timed with the reductions in the longitudinal length of the fascicles. Surprisingly, the magnitude of the transverse strains, as imaged within the ultrasound scanning planes, appears smaller than expected. However, the imaging methods preclude the measurement of strains perpendicular to the ultrasound scans. Fully 3D studies are needed to explore the exact nature of shape changes to the fascicles during contraction and to relate these to the mechanisms of muscle contraction.

## Figures and Tables

**Table 1:** Fourier coefficients for the medial gastrocnemius.

Parameter	MG		
	*c*_1_	*a*_1_	*o*_1_
*P*	−13.06 ± 0.20°	7.93 ± 0.19°	−1.4 ± 2.1°
*T*	7.59 ± 1.05 N m	21.30 ± 1.91 N m	252.7 ± 3.4°
${\hat{L}}_{f}$	1.00 ± 0.00	0.22 ± 0.02	29.7 ± 2.9°
${\hat{\lambda }}_{f}$	1.00 ± 0.00	0.017 ± 0.002	182.6 ± 9.2°
*θ*_*d*_	17.10 ± 0.45°	5.34 ± 0.46°	215.0 ± 2.8°
*θ*_*F*_	15.86 ± 0.27°	1.64 ± 0.14°	217.8 ± 3.6°
${\hat{L}}_{y}$	1.00 ± 0.00	0.023 ± 0.003	286.7 ± 13.2°
*B*_*p*_	146.27 ± 4.72	7.21 ± 0.48	38.4 ± 4.3°

Values are shown as mean ± SEM (*n* = 6) and are for the first harmonic of the Fourier series.

**Table 2:** Fourier coefficients for the lateral gastrocnemius.

Parameter		LG	
	*c*_1_	*a*_1_	o_1_
*P*	−13.06 ± 0.20°	7.93 ± 0.19°	−1.4 ± 2.1°
*T*	7.59 ± 1.05 N m	21.30 ± 1.91 N m	252.7 ± 3.4°
${\hat{L}}_{f}$	1.00 ± 0.00	0.19 ± 0.02	36.2 ± 5.4°
${\hat{\lambda }}_{f}$	1.00 ± 0.00	0.015 ± 0.001	209.3 ± 7.0°
*θ*_*d*_	9.23 ± 0.57°	2.54 ± 0.34°	219.7 ± 5.4°
*θ*_*F*_	11.40 ± 0.49°	1.62 ± 0.27°	216.6 ± 4.8°
${\hat{L}}_{y}$	1.00 ± 0.00	0.027 ± 0.003	262.1 ± 16.4°
*B*_*p*_	148.62 ± 4.17	7.22 ± 0.96	33.4 ± 5.5°

Values are shown as mean ± SEM (*n* = 6) and are for the first harmonic of the Fourier series.
